# A descriptive study of commercial herbal dietary supplements used for dyslipidemia—Sales data and suspected adverse reactions

**DOI:** 10.1002/ptr.7473

**Published:** 2022-05-07

**Authors:** Olta Allkanjari, Francesca Menniti‐Ippolito, Ilaria Ippoliti, Silvia Di Giacomo, Tito Piccioni, Annabella Vitalone

**Affiliations:** ^1^ Department of Physiology and Pharmacology ‘Vittorio Erspamer’ Sapienza University of Rome Rome Italy; ^2^ National Centre for Drug Research and Evaluation National Institute of Health Rome Italy; ^3^ Farmacia Piccioni Tito Rome Italy

**Keywords:** dyslipidemia, herbal dietary supplement, monacolin K, red yeast rice, sales data, suspected adverse reactions

## Abstract

Herbal dietary supplements (HDS) used for dyslipidemia represent a category of concern in Italy for suspected adverse reactions (ARs). However, we cannot estimate their safety, as we do not know their commercial profile. Sales data of HDS, and particularly, those used for dyslipidemia, were monitored for 2 years in two pharmacies of Rome. Meanwhile, spontaneous reports of suspected ARs potentially related to dyslipidemia supplements were collected by the Italian Phytovigilance System. The 50% of the total dietary supplements are herbal‐derived; the 9% of HDS are recommended for dyslipidemia. From our data, 113 different brands have claims for improving lipids profile and 91% of them are multiingredient preparations. Fifteen spontaneous reports of suspected ARs concerned HDS used, for dyslipidemia. The most frequent ARs were joint, abdominal, and muscles pain; vomiting; erythema and hematological disorders; nausea; and rhabdomyolysis. Our findings point out the limited compliance of commercial dyslipidemia‐HDS and scientific research about their intrinsic safety. A wide range of ingredients could not support the risk/benefit profile of the supplement. The variable compositions of HDS do not assure the safety, as they do not support the reproducibility of their pharmacological activities. This study could contribute to optimize consumer guidance about what they purchase and consume.

## 1 INTRODUCTION

In Italy, the use of food supplements containing botanicals is currently regulated by the Ministerial Decree of August 10, 2018 (Italian Ministry of Health, 2018). Accordingly, herbal dietary supplements (HDS) cannot claim a therapeutic activity but only nutritional or physiological effects. They can be placed on the market after a simple notification procedure addressed to the Italian Ministry of Health (MoH). Although it is always mandatory to conduct a quality control on the raw materials, no clinical safety trials are required. In light of the current safety issues supported by scientific evidence, the MoH has paid particular attention to herbal supplements containing, for example, curcumin and monacolin K (Mazzanti, Moro, Raschi, Da Cas, & Menniti‐Ippolito, 2017; Menniti‐Ippolito et al., 2020). Annex 1 of Decree of August 10, 2018, containing the list of permitted plants and their parts, accompanied where appropriate by additional provisions for use, has already been amended by a directorial decree dated July 26, 2019. The Decree has introduced special warnings concerning *Curcuma* genus (Italian Ministry of Health, 2019a). A maximum daily intake of 10 mg has been established for monacolin K and a new regulation of the European Commission (EC) has been suggested (European Commission, 2021), after the conclusions of the European Food Safety Authority (EFSA) about monacolin K from red yeast rice (RYR). Indeed, according to EFSA's scientific opinion, there is a significant safety concern of monacolins from RYR, when used as food supplements at the level of 10 mg/day. Moreover, individual cases have reported severe adverse reactions (ARs) even at the 3 mg/day intake (EFSA, 2018).

In the European Union (EU), so far, there is not a harmonized legislation for botanicals included in food supplements. The safety of HDS is a matter of discussion due to the lack of randomized clinical trials and postmarketing observational evidence (Allkanjari, 2021).

Cardiovascular diseases (CVDs) represent a growing global public health issue. As dyslipidemia is one of the risk factors that can lead to CVD, its management is one of the basic approaches to prevent CVD (Mach et al., 2020). Thus, clinical benefits are generally determined by lowering low‐density lipoprotein cholesterol (LDL‐C), total cholesterol, triglycerides (TGs), and increasing high‐density lipoprotein cholesterol (HDL‐C) plasma levels. According to the Guidelines of the European Society of Cardiology and the European Atherosclerosis Association (2019), healthy lifestyle habits regarding diet, smoking, alcohol, physical activity, etc. contribute to improve the overall lipid profile and should be promoted. Up to date, conversely of the clinical evidence of the pharmacological treatment, data on tolerability/safety of the dietary supplements/functional foods, and their possible usefulness (Mach et al., 2020) are scanty. In Italy, since 2002, the National Institute of Health (NIH), in cooperation with the MoH and the Italian Medicines Agency (AIFA), coordinates the Italian surveillance system (phytovigilance) that collects spontaneous reports of suspected ARs derived from herbal products and food supplements. The aim of Phytovigilance is to identify, to assess and eventually to prevent adverse effects deriving from dietary supplements, herbal products, galenic preparations, and other botanical‐derived products (Mazzanti et al., 2017).

In the EU as well as Norway, Liechtenstein, Switzerland, and Iceland, a centralized postmarketing vigilance approach is not yet implemented for HDS, which often boast pharmacological effects. Meanwhile, EudraVigilance, a large pharmacovigilance database that tracks suspected ARs to authorized medicines including herbal medicinal products, operates on behalf of the EU. However, Eudravigilance allows only the collection of data in aggregated manner. The Rapid Alert System for Food and Feed database detects risks in the food chain on behalf of the EC. Although it provides updated information on food public health warnings, its usefulness for food supplements is limited, as often does not report ARs due to dietary supplements (Kowalska & Manning, 2021).

It is not possible to estimate the real safety profile of dyslipidemia‐HDS, since we do not know their sales and the characteristics of commercial products. Indeed, HDS used in metabolic problems including dyslipidemia represents a category involved in a great number of suspected ARs, at least in Italy (Mazzanti et al., 2017; Vitalone et al., 2011). Monitoring sales data of dyslipidemia‐HDS would help to highlight the real risk/benefit profile of these products. Hence, the aim of this study is to monitor sales data, in territorial pharmacies, of HDS having specific health claims.

## 2 MATERIALS AND METHODS

### 2.1 Definition of dietary supplements, HDS, and dyslipidemia‐HDS

In our study, we consider as dietary supplements, all food supplements containing vitamins, minerals, amino acids, essential fatty acids, fibers, various plants, and herbal extracts according to the European Regulation (EC) No 1925/2006 on the addition of vitamins, minerals, and of certain other substances to foods (European Parliament, 2006). HDS include food supplements having at least one botanical ingredient. Dyslipidemia‐HDS refers to HDS having claims for lowering lipid values, according to the manufacturer labeling.

### 2.2 Data collection

Dietary supplement‐related sales, from databases of two pharmacies located in Rome (Italy), were monitored for 2 years. The location of pharmacy 1 is the historic center of Rome, while pharmacy 2 is located in a residential area. Data were collected from October 2018 up to September 2020.

A portable document format listing food supplements sold monthly was provided through WINGESFAR software from each pharmacy. In particular, the quantity and the brand names have been recorded. Only data of HDS, having health claims related to dyslipidemia, were included in the study.

Meanwhile, through the Italian Phytovigilance System, we collected and analyzed all the spontaneous reports of suspected ARs potentially related to antidyslipidemia supplements according the “reason of use” indicated in each report by citizens. Everyone, such as healthcare professional, patient, manufacturer, who observes a suspected AR, may send the report filling in an “ad hoc” form available on the websites of the involved institutions (NIH, MoH, AIFA). Since December 2018, reports were registered on the website www.vigierbe.it coordinated by NIH. The period of collection started from October 2018 to September 2020. All reports were registered in a database at the NIH.

### 2.3 Data preparation and descriptive analysis

Through Microsoft excel, a specific database was created. Data were monthly updated with new results obtained from each pharmacy. In particular, sales of the total dietary supplements, HDS, and dyslipidemia‐HDS were recorded and analyzed. Then, for each whole year, trends of sales of dietary supplements, HDS, and dyslipidemia‐HDS were assembled for both pharmacies and compared to each other. We consider as the first year the period ranging from October 2018 to September 2019 and as second year the period October 2019–September 2020.

Each of the dyslipidemia‐HDS has been analyzed for: composition (as described on the label), claims, and number of sales. The labels were obtained either from the manufacturer website (when reported) or other websites randomly selected using the product name as a keyword. A statistical descriptive procedure of the composition of each dyslipidemia‐HDS was performed. The distribution of the absolute and percentage frequencies of the data was analyzed. In particular, prevalence of the number and type (botanical and nonbotanical) of the components was considered. The occurrence of standardization, the plant material, and type of extract were also estimated. All the plant species found in the dyslipidemia‐HDS were clustered. The physiological functions of the plant materials were examined according to the guidelines of Italian MoH (Italian Ministry of Health, 2019b), which advise the use in the context of homeostasis model defined by the Council of Europe, as definitions of claims about botanicals are pending. The recurrence of other additional (nonbotanical) ingredients like vitamins, coenzymes, amino acids, probiotics, etc., was also recorded. The biological effects claimed for each product were assessed also by interfacing them with scientific literature data.

In relation to phytovigilance, a descriptive analysis was performed for all data regarding patient, supplement, clinical event, and reporter. The percentage of each HDS, reported for its suspected ARs, was carried out. The ARs were coded according to the Medical Dictionary for Regulatory Activities. Referring to the composition stated by the manufacturer, the frequency of each plant species and/or botanical ingredients found in the suspected supplements was also calculated.

## 3 RESULTS

### 3.1 Sales data

**FIGURE 1:**
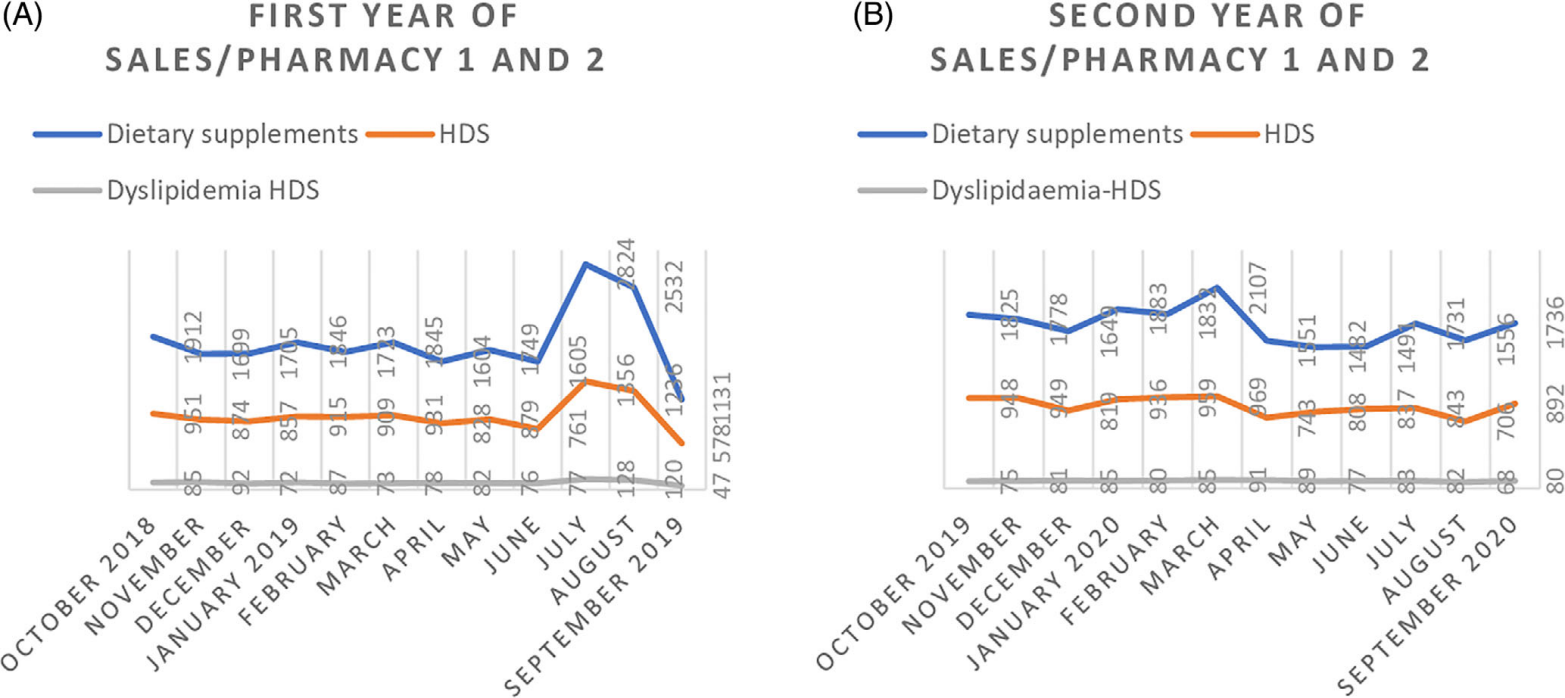
Sales from both pharmacies, during the first (a) and second (b) year

During 2 years of monitoring, 42,796 dietary supplements were sold by the two pharmacies selected, and 21,484 (50%) of them were HDS. Of the HDS, 1993 (9%) were dyslipidemia‐HDS. Referring to both pharmacies, the sales of each year indicate the same percentages of HDS (50%) and dyslipidemia‐HDS (9%). Descriptive sales data of both pharmacies are reported in Table 1. Monthly trend sales are described in Figure 1.

**TABLE 1:** Sales data reported annually for both pharmacies

	1° year			2° year		
	Dietary supplements	HDS	Dyslipidaemia‐HDS	Dietary supplements	HDS	Dyslipidaemia‐HDS
Pharmacy 1+2	22,175	11,075	1,017	20,621	10,409	976

### 3.2 Description of dyslipidemia‐HDS

Based on our data, 113 different brands have claims for improving lipids profile. Out of the (113) different dyslipidemia formulations, 9% (*n* = 10) contain a single ingredient, while 91% (*n* = 103) are multi‐ingredient preparations. The number of the ingredients in the same supplement, including botanicals and additional nonbotanical ones, range from two up to 14. In Table 2 are listed in alphabetic order all the different formulations of dyslipidemia‐HDS identified in the study. Alongside, the relative sales of each entry, referring to both pharmacies, and the number of the ingredients (botanicals and additional‐nonbotanicals) are registered. The phytochemical composition is reported without vehicles; daily doses are reported too, when indicated in the label.

**TABLE 2:** Dyslipidaemia‐HDS sold during the study

Number of sales	Brand name, pharmaceutical form, manufacturer	Phytochemical composition without vehicles, daily dose when reported	Number of ingredients
1	Active liver® 30 tablets, New Nordic	Milk thistle (*Silybum marianum* L.) extract (80% of silymarin, 250 mg); artichoke (*Cynara scolimus* L.) extract 13.5:1 (222 mg); curcuma (*Curcuma longa* L.) extract 10:1 (50 mg); choline 85 mg; black pepper (*Piper nigrum* L.) extract (40% of piperine, 10 mg)	5
2	Arkocapsule®Aglio 45 tablets, Arkopharma	Garlic (*Allium sativum* L.) bulb powder 900 mg	1
1	Aglio estratto oleoso® 50 capsules, Aboca	Garlic (*A. sativum*) bulbs oil extract 1:10	1
5	Alares® 20 tablets, Biotema	Lipoic acid, Lyc‐o‐lutein (lutein 20%, alginates, gum arabic, pea starch, Rosmarinus extract), polygonum (*Polygonum cuspidatum* Siebold & Zucc.) rhizome dry extract (98% of resveratrol)	4
31	Alenil Q® 30 tablets, Revalfarma	Red yeast rice 200 mg (monacolin K 10 mg); betaine 150 mg; coenzyme Q10, 50 mg; vitamin E 12 mg; vitamin B6 1.4 mg, folic acid 200 mcg; vitamin B12 2.5 mcg	7
107	Armolipid® 20, 30 tablets, Rottapharm	Red yeast rice (1.5% of monacolin, 3 mg); microalgae (*Haematococcus pluvialis*) dry extract (2.5% of astaxanthin, 0.5 mg); *Saccharum officinarum* (1.4% of policosanols, 10 mg); coenzyme Q10, 2 mg; folic acid 200 mcg	5
220	Armolipid plus® 20, 30, 60 tablets, Rottapharm	*Berberis aristata* dry extract 588 mg (85% of berberine 500 mg); red yeast rice 200 mg (1.5% of monacolin, 3 mg); microalgae (*H. pluvialis*) 20 mg (2.5% of astaxanthin, 0.5 mg); *S. officinarum* dry extract (90% of policosanols, 10 mg); folic acid 200 mcg; coenzyme Q10, 2 mg	6
1	Arteractiva® 36 tablets, Studio3farma	Capsicum powder; rosa canina dry extract 70%; *P. cuspidatum* dry extract (98% of resveratrol); black pepper dry extract 95%	4
19	Aterostar® 30 tablets, Stardea	Red yeast rice (1.5% of monacolin K); linear aliphatic alcohols (60% of octacosanol); niacin; green tea (*Camellia sinensis* L. Kuntze leaves) dry extract (40% of total polyphenols); vitamin E acetate (50% of alpha‐tocopherol acetate); pyridoxine hydrochloride; vitamin B12 (0.1% of cyanocobalamin); folic acid	8
12	Aterostar forte® 20 tablets, Stardea	Red yeast rice (3% of monacolin K, 10 mg); niacin; linear aliphatic alcohols (60% of octacosanol); green tea (*C. sinensis* (L.) Kuntze leaves) dry extract (40% of total polyphenols); vitamin E acetate (50% of alpha‐tocopherol acetate), olive (*Olea europaea* L. leaves) dry extract (15% of oleuropein); coenzyme Q10; pyridoxine hydrochloride; vitamin B12 (0.1% of cyanocobalamin); folic acid	10
14	Azelip® 20 sachets, Progine Farmaceutici	Red yeast rice 333.33 mg (monacolin 10 mg); inositol 1.50 g	2
24	Berberol®^a^ 30 tablets, PharmExtracta	*B. aristata* DC. Root dry extract 588 mg (85% of berberine, 500 mg); *S. marianum* (L) Gaertn. Fruits dry extract (silymarin 105 mg)	2
28	Berberol K®^a^ 30 tablets, PharmExtracta	*B. aristata* dry extract (berberine 500 mg); milk thistle (*S. marianum*) dry extract (silymarin 105 mg); red yeast rice extract(10 mg monacolin K)	3
4	Biostatine forte® 60 tablets, Pharmalife research	Red yeast rice dry extract (1.5% of monacolin K); artichoke (*Cynara scolymus*) leaves dry extract (2.5% of cynarine); citroflavonoids (60% of hesperidin)	3
6	Cadired 5® 36 tablets, Cadigroup	L‐carnitine 650 mg; red yeast rice 200 mg dry extract (5% of monacolin K, 10 mg); hawthorn (*Crataegus oxyacantha*) leaves 200 mg dry extract (1.8% vitexin, 3.6 mg); niacin 30 mg; microalgae (*H. pluvialis*) powder 2 mg (5% astaxanthin, 100 mcg); chromium 1.6 mg	6
1	Calip plus® 60 tablets, promopharma	Red yeast rice dry extract 334 mg (3% monacolin K, 10 mg); Biox save® (mixture of *O. europaea* L. fruit and *Vitis vinifera* L. fruit powder) 100 mg, coenzyme Q10 20 mg	4
16	Captolip® 24 tablets, Chemist's research	Red yeast rice 333.4 (3% monacolin K, 10 mg); policosanols 10 mg; coenzyme Q10 2 mg; thiamine (vit. B1) 1.1 mg; folic acid 200 mcg	5
2	Cardiofol®^a^ 30 cpr, named	Vitamin B6 1.4 mg; folic acid 0.2 mg; vitamin B12 2.5 mcg; *V. vinifera* fruit dry extract (titrated in flavonoids, anthocyanins and resveratrol) 40 mg	4
9	Cardiokolester 10® 30 tablets, Dymalife	Red yeast rice dry extract 200 mg (5% of monacolin K, 10 mg); curcuma (*C. longa* L.) rhizome dry extract 100 mg (95% of curcumin, 95 mg); sage (*Salvia miltiorrhiza* Bunge) root dry extract 70 mg; garlic (*A. sativum* L.) bulb dry extract 40 mg; astaxanthin 0,25 mg; pyridoxine hydrochloride (vitamin B6) 1.4 mg; chromium picolinate 40 mcg.	7
10	Cardiol forte® 30 tablets, U.G.A Nutraceuticals	Fish oil (412 mg), 75% omega‐3 (min. 63% EPA and DHA, 231 mg EPA and 115 mg DHA); red yeast rice 350 mg (3% of monacolin K, 10 mg; olive (*O. europaea* L.) fruit dry extract 50 mg (10% of hydroxytyrosol, 5 mg); coenzyme Q10 (100 mg); natural vitamin E 12 mg; folic acid 300 mcg; vitamin B12 2 mcg; *P. nigrum* L. fruit dry extract 1 mg (85% of piperine, 0.85 mg)	8
191	Cardiolipid 10® 30 tablets, 20 sachets, Shedir Pharma	Red yeast rice 200 mg (5% of monacolin K, 10 mg); fish oil 60 mg, 65% omega 3 (42% EPA and 27.6% DHA, 19.5 mg); alpha‐lipoic acid 30 mg; niacin, 27 mg; policosanol, 13 mg (60% of octacosanol, 7.8 mg; *P. cuspidatum* Siebold & Zucc. Rhizome 2.1 mg (98% of resveratrol, 2 mg); pantothenic acid 2 mg; folic acid 200 mcg; chromium picolinate 62 mcg; vitamin B12 2 mcg	10
16	Cardionam® 30, 60 tablets, Named	Artichoke (*C. scolymus* L.) leaves dry extract 500 mg (5% of caffeoylquinic acids); red yeast rice 200 mg (5% of monacolin K, 10 mg); banaba (*Lagerstroemia speciosa* L. Pers) leaves dry extract 75 mg (1% of corosolic acid); coenzyme Q10 50 mg; niacin 9 mg; vitamin B6 1.4 mg; vitamin B12 0.83 mcg; folic acid 110 mcg	8
4	Cardioqten 20® tablets, Carepharm	Indian berberi (*B. aristata* DC) bark dry extract (85% of berberine); red yeast rice dry extract (5% of monacolin K); coenzyme Q10; folic acid	4
27	Cardiostatin plus® 30 tablets, Criver Farmaceutici	Red yeast rice 200 mg (monacolin K 3 mg); sugar cane (*S. officinarum*) dry extract (policosanols 20 mg); beta‐sitosterol 75 mg; niacin 27 mg; zinc 10 mg; coenzyme Q10 10 mg; folic acid 200 mcg	7
1	Colber 30® tablets, Esserre pharma	Bergamot (*Citrus bergamia* Risso & A. Poit) fruit dry extract 200 mg (60% of flavonoids); phytosterols 120 mg, artichoke (*C. scolymus* L.) leaves dry extract 80 mg (2.5% of chlorogenic acid), vitamin C (L‐ascorbic acid) 20 mg	4
1	Colenorm plus®^a^ 30 tablets Inpha nutraceuticals	*P. cuspidatum* (resveratrol 20 mg); 20 mg policosanols (12 mg octacosanol); 333.3 mg red yeast rice (10 mg of monacolin K); 50 mcg chromium; 3.15 mg black pepper of which 2.99 mg piperine	5
5	Colesia oral gel® 20 sticks, IBSA Farmaceutici	Linseed (*Linum usitatissimum* L. oleum); apple annurcomplex (*Malus pumila* Miller cultivar Annurca) fruit dry extract; sterol esters titrated in sterols and stanols; red yeast rice (10% of monacolin K)	4
51	Colesia softgel 30®^a^ tablets, IBSA Farmaceutici	Sterol esters (59% of sterols and stanols); curcuma (*C. longa* L.) rhizome dry extract; red yeast rice (3% of monacolin K); olive (*Olea europea* L.) fruit dry extract titrated in polyphenols	4
4	Colesolv® 30 tablets, Glauber pharma	Red yeast rice 200 mg (1.5% of monacolin); berberis (*B. aristata* DC) bark dry extract 150 mg (98% of berberine); green tea (*C. sinensis* Kuntze) leaves dry extract 100 mg (50% of polyphenols); artichoke (*C. scolymus* L.) leaves dry extract 65 mg (2.5% of caffeoylquinic acids); alpha‐lipoic acid 50 mg; black pepper (*P. nigrum* L.) fruits dry extract 20 mg (95% of piperine), sugar cane (*S. officinarum* L.) dry extract (60% of octacosanol, 10 mg), coenzyme Q10 5 mg	8
11	Colest 500® 60 tablets, OTI (Officine Terapie innovative)	Berberis (*B. aristata* DC.) root dry extract 515 mg (98% of berberine, 505 mg); red yeast rice 334 mg (3% of monacolin K,10 mg); astaxanthin 5 mg; coenzyme Q10, 5 mg; folic acid 200 mcg; vitamin B12, 2 mcg	6
2	ColestaltQ10® 45 tablets, Rima Laboratori	Red yeast rice; gamma‐oryzanol; olive (*O. europea* L.) leaves dry extract; policosanols from rice (60% of octosanol); coenzyme Q10	5
370	Colestarmony plus® 20, 60 tablets, Biodue Spa	Red yeast rice 333 mg (3% of monacolin K, 10 mg and (2% of total polyphenols, 6.66 mg); berberine Bio‐Sol® 236 mg (berberine hydrochloride 47.2 mg); curcuma dry extract 50 mg (95% of curcumin, 47.5 mg); pomegranate dry extract 50 mg (40% of punicosides, 20 mg); coenzyme Q10, 2 mg	5
4	Colestat® 30 tablets, Difass	Red yeast rice 200 mg (1.5% of monacolin K); linear aliphatic alcohols 10 mg (60% of octacosanol); niacin 27 mg; green tea (*C. sinensis*) dry extract 144 mg (40% of polyphenols), vitamin E 20 mg; folic acid 300 mcg, vitamin B6 2 mg; vitamin B12 1 mcg	8
2	Colestereg® 24 tablets, Profar	Artichoke (*C. scolymus* L.) leaves dry extract (5–6% of caffeoylquinic acid); red yeast rice (3% of monacolin K); resveratrol from the root of *P. cuspidatum* Siebold & Zucc.	3
28	Colesterol act plus®^b^ 60 tablets, F&F srl.	Guggul (*Commiphora mukul*) resin 10 mg; *Coleus forskohlii* root 10 mg; red yeast rice 200 mg (5% of monacolin 10 mg); octacosanol 10 mg; beta‐sitosterol 50 mg; folic acid 200 mcg; Caigua (*Cyclantera pedata*) fruits 10 mg	7
2	Colestoil omega 3® 100 tablets, Aboca	Fish oil 1.25 g (min. 60% EPA and DHA, 750 mg); linseed oil 600 mg (50% of alpha‐linolenic acid, 300 mg); garlic bulb oil extract 150 mg; lemon (*Citrus limon*) essential oil 20.6 mg; vitamin E (12 mg of alpha‐tocopherol)	5
1	Colevis liquid analcolic® 500 ml Dr. Giorgini	Walnut (*Juglans regia*) fresh unripe fruits liquid integral extract; red yeast rice (1% of monacolin K), dandelion (*Taraxacum officinale*) root liquid integral extract; boldo (*Peumus boldus*) leaves liquid integral extract; artichoke (*C. scolymus*) leaves liquid integral extract; white horehound (*Marrubium vulgare*) aerial parts liquid integral extract; cleavers (*Galium aparine*) aerial parts liquid integral extract; milk thistle (*S. marianum*) fruit dry extract (40% of silymarin); rosemary (*Rosmarinus officinalis*) leaves liquid integral extract	9
7	Colex mu® 50 tablets, MU s.r.l.	Red yeast rice 360 mg (1.5% of monacolin, 1.26 mg); artichoke (*C. scolymus)* leaves 216 mg; *Plantago ovata* (ispaghula) seeds 71 mg; algae Klamath 71 mg	4
8	Coltrix®^a^ 30 tablets, Laboratori Legren	Policosanols from sugar cane 20 mg; red yeast rice 200 mg (1.5% of monacolin K); gamma‐oryzanol 100 mg; resveratrol 40 mg; coenzyme Q10, 10 mg; folic acid 100 mcg; vitamin E 10 mg	7
1	Corlipid® 30 tablets, Essecore	Red yeast rice 200 mg (1.5% of monacolin, 3 mg), green tea (*C. sinensis* Kuntze) leaves dry extract 100 mg (95% of polyphenols, 95 mg), policosanols from rice 10 mg (60% of octacosanol, 6 mg)	3
10	Desimet® 20 sachets, Gofarma	Inositol 1,500 mg; red yeast rice 333 mg (3% of monacolin K, 10 mg); Centellin® ‐ centella (*Centella asiatica* L.) leaves dry extract 60 mg; folic acid 200 mcg	4
17	Dislicol® 30 tablets, Deltha pharma	Red yeast rice 200 mg (1.5% of monacolin, 3 mg); policosanols 5 mg from sugar cane (90% of octacosanol); artichoke150 mg; resveratrol 20 mg; folic acid 0.20 mg	5
1	Epadx® 40 tablets AVD reform	N‐acetyl cysteine 400 mg; *S. marianum* Gaertn fruit dry extract 400 mg (132 mg silymarin fitosoma®; Fumaria dry extract 200 mg; SAMe (S‐Adenosyl‐methionine) 200 mg; Cultavit®: natural B complex from buckwheat (*Fagopyrum esculentum* Moench) whole fruit 150.5 mg (0.31 mg of B1, 0.5 mg of B2, 5.4 mg of B3, 2 mg of B5, 0.31 mg B6, 68 mcg of folic acid, 0.6 mcg B12, 16.5 mcg of biotin); zinc gluconate 10 mg	6
1	Epatoguna® 32 tablets, Guna	Freeze‐dried pork liver; choline bitartrate; green tea (*C. sinensis* L. Kuntze) leaves dry extract (40% of epigallocatechin gallate (EGCG)	3
5	Equitrig® 30 tablets, Trendfarma	Red yeast rice 334 mg (monacolin K 10 mg); *Cassia nomame* (*Cassia mimosoides* L. var *nomame* Makino) 200 mg (16 mg of catechins); niacin 24 mg; policosanols 10 mg (octacosanol 0.5 mg); folic acid 200 mcg	5
26	Esterol 10®^a^, 20 tablets, Laborest	Red yeast rice (5% of monacolin K, 10 mg); green tea (*Camelia sinensis* L. Kuntze) leaves dry extract 100 mg (40% of EGCG); coenzyme Q10 20 mg; microalgae (*H. pluvialis* Flotow, thallus (astaxanthin 2 mg); resveratrol (*P. cuspidatum* Siebold & Zucc.) rhizome 20 mg; quercetin 50 mg; vitamin D 5 mcg; vitamin K2 75 mcg; folic acid 200 mcg; selenium 83 mcg	10
5	Eufortyn colesterolo® 30 tablets, Scharper	Dried bergamot juice; coenzyme Q10; choline; zinc	4
1	Experid‐250®^a^ 50 tablets, Biotema	*Citrus sinensis* fruit (hesperidin), pomegranate (*Punica granatum* L.) fruit (20% of ellagic acid); bilberry (*Vaccinium myrtillus* L.) fruit dry extract (1% of anthocyanosides	3
1	Ezimega®^a^ 20 tablets, Alfasigma	Fish oil (65% of omega 3,604 mg, 165 mg EPA and 121 mg DHA); red yeast rice 200 mg (1.5% of monacolin, 3 mg); L‐carnitine 147 mg; resveratrol 10 mg; coenzyme Q10, 10 mg; policosanols from sugar cane (*S. officinarum*) 10 mg; vitamin B6, 3 mg; vitamin B12 2.5 mcg	8
1	Ezimega 3® 20 tablets, Alfasigma	Red yeast rice (5% of monacolin K), *Lactobacillus plantarum* ECGC 13110402, resveratrol from *P. cuspidatum* Siebold & Zucc. rhizome dry extract, coenzyme Q10, folic acid	5
77	Ezimega plus®^a^ 20 tablets, Alfasigma	Fish oil 651 mg of omega 3 (100 mg of DHA, 150 mg of EPA); coenzyme Q10, 10 mg; resveratrol 10 mg; folic acid 300 mcg; vitamin B12 2.5 mcg; vitamin B6 3 mg; red yeast rice 417 mg (3% of monacolin K, 10 mg); policosanols from sugar cane (*S. officinarum* L.) 10 mg	8
29	Faros® 30 tablets, Fidia Farmaceutici	Red yeast rice (5% of monacolin K, 10 mg) Revifast® (*P. cuspidatum* Siebold & Zucc. rhizome extract) (min. 30% of resveratrol)	2
2	Farostin® 20 tablets, Fidia	Berberis (*B. aristata* DC) branches bark dry extract (85% of berberine chloride); Revifast® (*P. cuspidatum* Siebold & Zucc. rhizome extract) (min. 30% of resveratrol); Astragalus (*Astragalus microcephalus* Willd.) root dry extract (70% of polysaccharides)	3
1	Fitobiostatin® 30 tablets, Fitobios	Red yeast rice (1.5% of monacolin K); artichoke (*C. scolymus* L.) leaves dry extract (5% of chlorogenic acid); Nopal cladodes (*Opuntia ficus‐indica* Mill.) powder; vitamin E; gamma oryzanol; coenzyme Q10; folic acid	7
3	Fito‐omocisteina® 60 tablets, Solgar	Trimethylglycine (*Beta vulgaris*) 500 mg; vitamin B6 (25 mg of pyridoxin hydrochloride, 3 mg of pyridoxal 5‐phosphate); vitamin B12, 250 mcg; folic acid 200 mcg; powder Phyto2x (beta‐carotene); vitamin C	6
38	Glicemarmony® 30 tablets, Biodue Spa	Berberine Bio‐Sol® (*B. aristata* DC) bark dry extract 1,000 mg (20% of berberine hydrochloride, 200 mg); white mulberry (*Morus alba* L.) dry extract 200 mg (2% of 1‐deoxynojirimycin, 4 mg); curcuma (*C. longa* L.) root dry extract 100 mg (95% of curcumin 95 mg)	3
3	Iceflox® 20 tablets, Cetra Italia	Pineapple (*Ananas comosus* Merr.) stem and fruits 2,500 GDU/g (400 GDU of bromelain, 160 mg); quercetin 150 mg; curcuma (*C. longa* L.) root dry extract (95% of curcumin, 200 mg); green tea (*C. sinensis* L. Kuntze) leaves dry extract (98% of total polyphenols and 40% of EGCG, 70 mg); black pepper (*P. nigrum* L.) fruit dry extract (95% of piperine, 2.6 mg)	5
2	Insulipid®^b^ 30 tablets, Piemme Pharmatech	Berberine 200 mg; L‐arginine 200 mg; L‐carnitine 150 mg; *Spirulina platensis* 100 mg; red yeast rice 150 mg; *Euphrasia superba* (krill) 100 mg; chromium 20 mcg	7
12	Kilocal colesterolo®^a^ ^,^ ^b^ 15, 30 tablets, Pool Pharma	Red yeast rice 200 mg (monacolin K, 10 mg); fenugreek 100 mg (*Trigonella foenum graecum* L.) seed dry extract titrated in saponins; lespedeza (*Lespedeza capitata* Mich.) leaves and whole plant dry extract 100 mg, titrated in flavones; olive (*O. europaea* L.) leaves dry extract 100 mg (oleuropein 6 mg); white mulberry (*M. alba* L.) leaves dry extract 100 mg; berberine powder (*B. aristata* DC.) bark dry extract 50 mg (85% of berberine, 42.5 mg); willow (*Salix alba* L.) bark dry extract 50 mg (20% of salicin, 10 mg); banaba (*L. speciosa* L.) leaves dry extract; coleus (*C. forskohlii*) root dry extract 25 mg (10% of forskolin); coenzyme Q10, 5 mg; chromium picolinate 200 mcg; folic acid 200 mcg	12
4	Klamath RX Max® 60, 180 tablets, Nutrigea	Microalgae Klamath RW®max (*Aphanizomenon Flos Aquae*)	1
34	Kogi plus®^a^ 24 tablets, Bromatech	Coenzyme Q10, 20 mg; green tea (*C. sinensis* L.) leaves dry extract 60 mg; red yeast rice 200 mg (monacolin 10 mg); fruit extra‐virgin olive oil (*O. europea* L.) titrated in spinacetin; DL‐alfa tocoferolo	5
16	Koginet® 24 tablets, Bromatech	Annurca apple (*M. pumila* Mill. cultivar *annurca*) fruit dry extract 400 mg; l‐teanina 100 mg; probiotic mixture (*Lactobacillus acidophilus* LA‐14, *L. plantarum* LP‐115); gum Arabic (*Acacia Senegal* Wiud.) powder 30 mg; vitamin Bl, 1.1 mg	6
15	Kolestina 10 complex® tablets, Cosval Group	Red rice (*Oryza sativa* L.) seed dry extract fermented with *Monascus purpureus* Went. sporophore (37.3%) 200 mg (10% of monacolin K, 10 mg); Venere black rice 100 mg (*O. sativa* L.) seed dry extract 18.5% (25% of anthocyanosides, 25 mg); guggul (*C. mukul* Hook) resin dry extract 13%, 70 mg (10% of guggul sterols, 7 mg); sugar cane (*S. officinarum* L.) juice dry extract 3.7%, 20 mg (60% of policosanols, 12 mg)	4
2	Lactoflorene colesterolo® 30 tablets, Montefarmaco	Red yeast rice (3% of monacolin K, 10 mg); *Bifidobacterium longum* BB536® (ATCC BAA‐999) 1 billion UFC; coenzyme Q10, 20 mg; niacin 16 mg	4
1	Lecitina di soia® 400 g, Marco Viti	Soya lecithin (*Glycine max*); vitamin E acetate; vitamin B6 hydrochloride	3
8	Lecitina di soia® 45 tablets, Arkopharma	Soya lecithin (*Glycine max* (L.) Merr.)‐soya oil 1,650 mg	1
8	Levelipduo® 20 tablets, 14 sticks, Laboratori Guidotti	Sterols of vegetal origin 800 mg; red yeast rice 167 mg (3% of monacolin k, 5 mg); niacin 27 mg; policosanols 10 mg (60% of octacosanol)	4
3	LFP colesttab®^a^ 30 tablets 5 mg, Unifarco	Red yeast rice 334 mg (3% of monacolin k, 10 mg); coenzyme Q10, 100 mg; *H. pluvialis* Flotow thallus (2 mg of astaxanthin); policosanols of sugar cane (*S. officinarum* L.) juice 20 mg; folic acid 400 mcg	5
32	Lfp colesttab®^a^ 30 tablets 10 mg, Unifarco	Red yeast rice 210 mg (5% of monacolin K, 10 mg); coenzyme Q10, 100 mg; *H. pluvialis* flotow thallus (1 mg of astaxanthin); policosanols from sugar cane (*S. officinarum* L.) juice 10 mg; folic acid 200 mcg	5
19	Lipalt®^a^ 30 tablets, Farmacrimi	*C. bergamia* Risso & Poit. fruit 250 mg; red yeast rice 167 mg (3% of monacolin K); policosanols from sugar cane (*S. officinarum* L.) 10 mg; coenzyme Q10, 2 mg	4
18	Lipocol plus® 30 tablets, Laboratori Nutriphyt	Red yeast rice (5% of monacolin K); bergamot dry extract of fruit juice (25–28% of flavonoids); pine (*Pinus massoniana*) bark dry extract; vitamin E; vitamin B3; vitamin B12; vitamin B6; vitamin B1; folic acid	9
1	Liposan forte® 60 tablets Salugea	Red yeast rice 334 mg (3% of monacolin K, 10 mg); barberry (*Berberis vulgaris* L.) root bark dry extract 266 mg (98% of berberine, 260 mg); milk thistle (*S. marianum* Gaertn.) fruits dry extract 100 mg (80% of silymarin, 80 mg); rosemary (*R. officinalis* L.) leaves dry extract 1:4, 100 mg	4
2	Liposcudil®^a^ 30 tablets, Piam farmaceutici Spa	Policosanols from rice 10 mg; red yeast rice 200 mg (1.5% of monacolin K, 3 mg); phytosterols (95%) 50 mg; Extramel®‐melon pulp (*Cucumis melo*) 5 mg, *V. vinifera* seeds extract (95% of proanthocyanidins); Olivex®‐olive polyphenols 10 mg; folic acid 0.2 mg	7
5	Liposcudil BBR® 30 tablets, Piam Farmaceutici	Red yeast rice 100 mg (3% of monacolin K, 3 mg); *B. aristata* 572 mg (87.4% of berberine, 500 mg); chromium 40 mg; coenzyme Q10, 30 mg; folic acid 200 mcg	5
82	Liposcudil plus® 30 tablets, sachets, Piam Farmaceutici	Red yeast rice 333 mg (3% of monacolin K, 10 mg); coenzyme Q10, 30 mg	2
17	Long life olio germe grano® 60 tablets, Longlife	Wheat germ (*Triticum aestivum* L.) oil 1,500 mg: 62% of polyunsaturated fatty acids (930 mg), of which 40% of linoleic acid (omega‐6, 372 mg) and 10% of oleic acid (omega‐9, 150 mg); 0.15% of vitamin E 2.2 mg	1
4	Long life riso rosso fermentato® 60 tablets, Longlife	Red yeast rice 334 mg (3% of monacolin K, 10 mg); coenzyme Q10, 30 mg	2
12	Lopiglik®^a^ 20 tablets, AkademyPharma	Berberis (*B. aristata* D.C.) bark dry extract 531.25 mg (85% of berberine); white mulberry (*M. alba* L.) leaves dry extract (4 mg of deoxynojirimycin); red yeast rice 220 mg (1.5% of monacolin K, 3.3 mg)	3
11	Lopiglik plus® 20 tablets, Akademypharma	Berberis (*B. aristata* DC.) bark dry extract 531.25 mg (85% of berberine); red yeast rice 220 mg (1.5% of monacolin K, 3.3 mg); white mulberry (*M. alba* L.) leaves dry extract (2% of deoxynojirimycin, 4 mg); cholecalciferol powder (vitamin D3) 15,00 mcg (600 U.I.)	4
1	Lovachole® 30 tablets, Nalkein pharma	Red yeast rice 200 mg (5% of monacolin K, 10 mg); linseed oil (*L. usitatissimum* L.) 100 mg; alpha lipoic 50 mg; niacin 20 mg; zinc oxide 12.5 mg; vitamin B12 2.5 mcg; chromium picolinate 50 mcg	7
3	Loxicor® 30 tablets, 20 sachets, Logidex	Pine (*Pinus ssp*.) stems (99% of phytosterols); red yeast rice (5% of monacolin K); coenzyme Q10	3
5	Monalip® 30 tablets, biogroup	Red yeast rice 334 mg (3% of monacolin, 10 mg); coenzyme Q10, 10 mg	2
11	Nocol plus® 30 tablets, Princeps	Red yeast rice 200 mg (monacolin K 10 mg); total sterols 150 mg (beta‐sitosterol 112.5 mg); coenzyme Q10, 50 mg; policosanols 10 mg (octacosanol 6 mg)	4
1	Nocolesteno® 30 tablets, Elmafarma srl	Red yeast rice 200 mg (min. 1.5% of monacolin k, 3 mg); coenzyme Q10, 80 mg	2
5	No‐colest omegasol formula potenziata®, 40 tablets, Specchiasol	Bergamot fruit dry extract 42 mg (polyphenols 25 mg); red yeast rice 200 mg (5% of monacolin K, 10 mg); algae oil 46 mg (35% of natural DHA, 16 mg); coenzyme Q10, 5 mg	4
18	Normolip 5® 30 tablets, Esi	Red yeast rice 200 mg (5% of monacolin K, 10 mg); gamma‐oryzanol 90 mg; coenzyme Q10, 10 mg; policosanols 5 mg; chromium 200 mcg	5
6	Nova lipid plus® 30 tablets, Nova Argentia	Red yeast rice (5% of monacolin); olive (*O. europea* L.) leaves extract; artichoke (*C. scolymus* L.) leaves extract; grapevine (*V. vinifera* L.) seeds and leaves extract; coenzyme Q10; calcium pantothenate; vitamin B6; vitamin B2; vitamin B1; folic acid; chromium picolinate; vitamin H (biotin); vitamin B12	13
23	Nurvast® 30 tablets, Coohesion pharma	Annurca apple (*M. pumila* Miller) dry extract 800 mg (0.03% of Chlorogenic acid, 0.24 mg; 0.15% of phlorizin, 1.2 mg; 0.04% of procyanidin B2, 0.32 mg; 0.5% of ursolic acid, 4 mg)	1
1	Nurvast plus® 30 tablets, Coohesion pharma	Annurca apple (*M. pumila* Miller) dry extract 400 mg (0.03% of Chlorogenic acid, 0.12 mg; 0.15% of phlorizin, 0.6 mg; 0.04% of procyanidin B2, 0.16 mg; 0.5% of ursolic acid, 2 mg); policosanols from sugar cane (*S. officinarum* L.) 20 mg (60% of octacosanol, 12 mg)	2
3	Nutricol®^b^ 30, 120 tablets, Nutrigea	Psyllium (*P. ovata* Forsk.) husk 450 mg; dandelion (*T. officinale* DC) root 150 mg; *Bifidobacterium Bifidum* (DSM 25565) 16.2 billion UFC; *Aloe ferox* Mill. juice 105 mg; chinese rhubarb (*Rheum palmatum* L. var. *tanguticum* Max) rhizome 105 mg; microalgae Klamath RW® max (*Aphanizomenon Flos Aquae*) 90 mg; EnzyMax® (fermented maltodextrin) 90 mg; *B. aristata* DC. bark dry extract (8% of berberine); cascara sagrada (*Rhamnus purshiana* DC) bark dry extract 60 mg (18–20% of cascaroside A, 10.8 mg); *Echinacea purpurea* L. Moench. dry extract 30 mg (4% of total polyphenols, 1.2 mg); milk thistle (*S. marianum* L. Gaertn.) fruits dry extract 30 mg (80% of silymarin, 24 mg; 30% of silibinin and isosilibinin, 9 mg); ginger (*Zingiber officinale* Roscoe) rhizome 30 mg; fennel (*Foeniculum vulgare* Miller.) fruits 30 mg; bamboo (*Bambusa spp*.) sprouts dry extract 30 mg (75% of silicium, 22.5 mg)	14
2	Olivis liquido® 50 ml, Dr. Giorgini	Olive (*O. europaea*) leaves liquid integral extract; hawthorn (*C. oxyacantha*) flowers and leaves liquid integral extract; mistletoe (*Viscum album*) aerial parts liquid integral extract; shepherd's purse (*Capsella bursa‐pastoris*) aerial parts liquid integral extract; fumaria (*Fumaria officinalis*) aerial parts liquid integral extract	5
10	Omegadin plus retard® 30 tablets, GD tecnologie interdisciplinari farmaceutiche srl	Ahiflower® oil (*Buglossoides arvensis*) seeds (60% of omega‐3, 15% of omega‐6, 6% of omega‐9); olive oil; red orange complex (R.O.C.) Sicily red orange extract titrated; vitamin C; vitamin E; selenium 25 mcg	6
22	Omega 3 age® 45 tablets, Fitobios	Linseed oil (*L. usitatissimum* L.); grapevine (*V. vinifera* L.) seeds dry extract (75% of polyphenols); olive fruits (*O. europaea* L.) dry extract (10% of hydroxytyrosol); vitamin E	4
27	Omega formula® 80 tablets, Guna	Baobab (*Adansonia digitata* L.) seed 1,500 mg (5.4 mg of omega 3, 89.25 mg of omega 6, 85.95 mg of omega 9, 1.50 mg of polyphenols, 5.10 mg of sterols; red yeast rice powder 666.66 mg (monacolins 10 mg); vitamin B6, 3 mg; folic acid 300 mcg	4
4	Oxolipid® 30 tablets, Sanamedica group	Red yeast rice (5% of monacolin K); mangosteen (*Garcinia mangostana* L.) fruit pulp dry extract (40% of mangostins)	2
1	Pantavis 600 lipoico®, 20 tablets, biodelta	Alpha lipoic acid; berberine; gymnema (*Marsdenia sylvestris* (Retz.) P.I. Forst.) leaves dry extract (25% of gymnemic acid); chromium picolinate	4
5	Polidal 75®^a^ 20 tablets, Ghimas	*Fallopia japonica* (Japanese knotweed) (*P. cuspidatum*) extract (75 mg of polydatin)	1
8	Polidase® 30 tablets, Sherman tree nutraceuticals	Fallopia japonica (*P. cuspidatum*) dry extract (98% of polydatin, 160 mg);	1
1	Profito ST® drops 50 ml, Qantiqa	Mistletoe 7.50 mg; rosemary 7.50 mg; rhubarb 7.50 mg; black radish (*Raphanus sativum*) 7.50 mg	4
23	Redulip® 60 tablets, ForFarma	Artichoke (*C. scolymus* L.) leaves dry extract (5% of chlorogenic acid); red yeast rice (3% of monacolin K); *Fallopia japonica* Houtt Ronse Dec. rhizome (resveratrol); coenzyme Q10; chromium picolinate; vitamin B6; folic acid; vitamin B12	8
4	Resveratrox® 60 tablets, Solgar	*P. cuspidatum* Sieb. et Zucc. rhizome dry extract 400 mg (50% of resveratrol, 200 mg)	1
2	Revidox+® 60 tablets, Paladin pharma	Stilvid®‐grapevine (*V. vinifera* L.) fruit and seed dry extract 133 mg titrated; pomegranate (*P. granatum* L.) fruit dry extract 125 mg; selenium 34 mcg; vitamin C, 12 mg; zinc 1.5 mg; vitamin B2, 1.4 mg	6
2	Riscol 5® 30 tablets, Errekappa euroterapici spa	Red yeast rice 200 mg (monacolin K 10 mg); policosanols 20 mg (octacosanol 12 mg); resveratrol 40 mg	3
15	Riscol plus® 30 tablets, Errekappa Euroterapici	Fish oil 800 mg (40% of EPA, 288 mg and 30% of DHA, 244 mg); Bergavit® (*C. bergamia* Risso & Poit) fruit 400 mg, red yeast rice 200 mg (5% of monacolin K, 10 mg); Cromax® (chromium, 20 mcg)	4
2	Ritenil depura® 30 tablets, Syrio	Kale (*Brassica oleracea* L.) juice powder 300 mg; choline bitartrate 90 mg; desmodium (*Desmodium adscendens* Sw. DC.) leaves dry extract 200 mg; milk thistle (*S. marianum* L. Gaertn.) fruit dry extract 100 mg	4
3	Sincrolipid® 60 tablets, UP pharma	Policosanols; berberine; red yeast rice; cassia nomame; astaxanthin; coenzyme Q10; folic acid	7
2	Tegradoc® 30 tablets, Doc generic	Berberine 200 mg; red yeast rice 100 mg (monacolin K 3 mg); chitosan 100 mg; coenzyme Q10, 10 mg	4
2	Tirecol® 30 tablets, Fera Pharma	Selenium 83mcg; myo‐inositol 400 mg; vitamin D3 5 mcg; red yeast rice 334 mg (monacolin K 10 mg)	4
3	Tokaber plus® 30 tablets, Polifarma	*O. europea* L. leaves dry extract; Italian bergamot juice; choline; zinc	4
1	Trigofien® 60 tablets, Sarandrea	Fenugreek (*Trigonella foenum‐graecum* L.) seeds dry extract 1:4, 2,400 mg; fenugreek (*Trigonella foenum‐graecum* L.) seeds powder 540 mg	1
2	Trixy® 28 tablets, Nathura	*B. aristata* DC. bark dry extract 588 mg (85% of berberine); *Elaeis guineensis* Jacq. oleum dry extract 143 mg (21% of tocotrienols); coffee (*Coffea arabica*) without caffeine seed dry extract 67 mg (45% of chlorogenic acid)	3
1	Viridian EPA&DHA® liquid vegan	Marine algae (*Schizochytrium* spp.) oil; chia seed (*Salvia hispanica*) oil; natural orange oil; vitamin E	4
13	Zeta colest® 30 tablets, Erbozeta	Red yeast rice (3% of monacolin), milk thistle (*S. marianum*) fruits dry extract (80% of silymarin); guggul (*C. mukul* Hook) resin dry extract (10% of guggulipids and 2.5% of guggulsterones; sugar cane (*S. officinarum* LJ.) juice (98% of policosanols and 60% of octacosanols)	4

*Note*: The phytochemical composition obtained from other sources different from the manufacturer website is reported in gray colour.

^a^ The label reports the active ingredients and/or phytochemicals without percentage.

^b^ The label describes only the kind of phytoextract.

### 3.3 Description of the botanicals and the additional ingredients

Seventy‐one plant species, 17 isolated phytochemicals, and 31 additional nonbotanical ingredients were clustered from dyslipidemia‐HDS. In Table 3 are reported all the plant species in decreasing order of prevalence, along with the plant materials and the types of extract found in commercial dyslipidemia‐HDS. Furthermore, the markers with the relative range reported and the physiological functions (pending claims), according to the MoH, are also described.

**TABLE 3:** Plant species found in dyslipidaemia‐HDS, and their relative descriptions

Plant^a^	Plant material^b^	Type of extract^b^	Prevalence in dyslipidaemia‐HDS No. (%)^c^	Marker/range (%)	Physiological function according to the MoH^d^
*Oryza sativa* fermented with *Monascus purpureus*	Seed fermented with sporophore	Dry extract	72 (64%)	Monacolin K, 1–10%	Substance/nutrient^e^
*Berberis aristata* DC.	Root, bark, branches bark	Dry extract, powder	19 (17%)	Berberine, 8–98%	*Cortex ex ramis*: Regular function of the cardiovascular system. Digestive function. Hepatic functionality. Regularity of intestinal transit. Functionality of the digestive system.
*Saccharum officinarum* L.	Stem (cane)	Dry extract, juice	13 (12%)	Policosanols, 1.4–98% Octacosanol, 60–90%	*Succus*: Digestive function. Fluidity of bronchial secretions
*Olea europea* L.	Fruit, leaf	Dry extract, powder, oil, liquid integral extract	13 (12%)	Oleuropeina, 6–15% Hydroxytyrosol, 10% Spinacetin^f^	*Folium*: Carbohydrate and lipid metabolism. Normal blood circulation. Regularity of blood pressure. Antioxidant.
*Fallopia Japonica* (Houtt.) Ronse Dec. (*Polygonum cuspidatum* Siebold & Zucc.)^g^	Rhizome	Dry extract	12 (11%)	Resveratrol, 30–98% Polydatin, 98%	*Radix*: Antioxidant. Fluidity of bronchial secretions. Detoxification of the organism. Body fluids drainage. Regularity of the menstrual cycle. Regular function of the cardiovascular system. Tonic (physical, mental fatigue).
*Cynara scolymus* L.	Leaf	Dry extract, liquid integral extract	12 (11%)	Cynarine, 2.5% Caffeoylquinic acids, 2.5–6% Chlorogenic acid, 2.5–5%	*Folium*: Digestive function. Hepatic functionality. Elimination of intestinal gas. Detoxification of the organism. Lipid metabolism. Antioxidant.
*Silybum marianum* (L.) Gaertn.	Fruit	Dry extract	9 (8%)	Silymarin, 33–80% Silibin/isosilibin, 30%	*Fructus, tegumen seminis*: Digestive function. Hepatic functionality. Detoxification of the organism. Antioxidant. Carbohydrate metabolism.
*Camellia sinensis* (L.) Kuntze	Leaf	Dry extract	8 (7%)	Polyphenols, 40–98% Epigallocatechin gallate, 40%	*Folium*: Body fluids drainage. Body weight balance. Normal intestinal function. Tonic (physical, mental fatigue). Antioxidant.
*Citrus bergamia* Risso & Poit. *Citrus aurantium* var. *bergamia* (Risso) Brandis.^g^	Fruit	Dry extract, juice dry extract	7 (6%)	Flavonoids, 25–60% Polyphenols, 59.5%	ND^h^
*Curcuma longa* L.	Rhizome	Dry extract	6 (5%)	Curcumin, 95%	*Rhizoma*: Joint function. Antioxidant. Improvement of menstrual cycle disorders.
*Haematococcus pluvialis* Flotow	Thallus	Dry extract, powder	6 (5%)	Astaxanthin, 2.5–5%	*Thallus*: Antioxidant.
*Vitis vinifera* L.	Fruit, seed, leaf	Dry extract, powder	6 (5%)	Polyphenols, 75% Proanthocyanidins, 95% Flavonoids^g^ Resveratrol^g^ Anthocyanins^g^	*Folium, semen*: Microcirculation functionality (heavy legs). Antioxidant. Regular function of the cardiovascular system.
*Piper nigrum* L.	Fruit	Dry extract	6 (5%)	Piperine, 40–95%	*Fructus, oleum‐resina, oleum*: Digestive function. Regularity of intestinal transit. Regular gastrointestinal motility and gas elimination. Antioxidant. Regular function of the cardiovascular system.
*Allium sativum* L.	Bulb	Dry extract, powder, oil extract	4 (4%)	ND	*Bulbus*: Regular function of the cardiovascular system. Antioxidant. Triglycerides and cholesterol metabolism. Regularity of blood pressure. Bronchial secretions fluidity. Nose and throat wellness. Digestive function.
*Linum usitatissimum* L.	Seed	Oil extract	4 (4%)	Alpha‐linolenic acid, 50%	*Semen, tegumen seminis*: Regularity of intestinal transit. Normalized stool volume and consistency. Emollient and soothing action (digestive system). Modulation/limitation of nutrient absorption. Lipid metabolism. *Oleum*: Lipid metabolism. Integrity and functionality of cell membranes.
*Malus pumila* Mill. cv *annurca*	Fruit	Dry extract	4 (4%)	Chlorogenic acid, 0.03% Phlorizin, 0.15% Procyanidin B2, 0.04% Ursolic acid, 0.5%	ND
*Morus alba* L.	Leaf	Dry extract	4 (4%)	1‐deoxynojirimycin, 2%	*Cortex ex radicibus, folium, fructus*: Regularity of the intestinal transit. Body fluids drainage. Fluidity of bronchial secretions. Carbohydrate metabolism. Regularity of blood pressure. Functionality of urinary tract.
*Rosmarinus officinalis* L.	Leaf	Dry extract, liquid integral extract	4 (4%)	ND^h^	*Aetheroleum, folium*: Digestive function. Hepatic functionality. Regular gastrointestinal motility and gas elimination. Antioxidant. Regular function of the cardiovascular system.
*Aphanizomenon flos‐aquae*	ND	ND	3 (3%)	ND	*Bacteria*: Mood balance.
*Commiphora mukul* (Hook. Ex Stocks) Engl.	Resin	Dry extract	3 (3%)	Guggulsterols, 10% Guggulipids, 10% Guggulsterones, 2.5%	*Oleum‐gummi‐resina*: Lipid metabolism. Skin regeneration and functionality. Body weight balance.
*Punica granatum* L.	Fruit	Dry extract	3 (3%)	Ellagic acid, 20% Punicosides, 40%	*Fructus*: Antioxidant. *Pericarpum*: Digestive system functionality. Regularity of intestinal transit
*Cassia mimosoides* var. *nomame* (Siebold) Makino *Chamaecrista nomame* (Sieber) H. Ohashi^g^	ND	ND	2 (2%)	Catechins, 8%	*Folium, fructus*: Triglycerides and cholesterol metabolism. Body weight balance.
*Coleus forskohlii* (Willd.) Bricq.	Root	Dry extract	2 (2%)	Forskolin, 10%	*Radix, tuber*: Regular functionality of the cardiovascular system. Regularity of blood pressure. Functionality of the upper respiratory tract. Digestive function. Body weight balance.
*Crataegus oxyacantha* *Crataegus laevigata* (Poir.) DC.^g^	Leaf, flowers	Dry extract, liquid integral extract	2 (2%)	Vitexin, 1.8%	*Folium, flos*: Regular functionality of the cardiovascular system. Antioxidant. Relaxation and mental wellness. Regularity of blood pressure.
*Fumaria officinalis* L.	Aerial parts	Dry extract, liquid integral extract	2 (2%)	ND	*Herba* cum *floribus, summitas*: Hepatobiliary and digestive function. Detoxification of the organism. Skin regeneration and functionality (wellness).
*Lagerstroemia speciosa* (L.) Pers.	Leaf	Dry extract	2 (2%)	Corosolic acid, 1%	*Folium*: Digestive system functionality. Regularity of intestinal transit.
*Plantago ovata* Forssk.	Seed, husk	ND	2 (2%)	ND	*Semen, tegumentum seminis*: Regularity of the intestinal transit. Emollient and soothing effect (digestive system). Modulation/limitation of nutrient absorption. Lipid and carbohydrate metabolism. Stool normal volume and consistency. Prebiotic effect.
*Glycine max* (L.) Merr.	ND	Oil	2 (2%)	ND	*Semen, semen germinatus*: Lipid metabolism. Improvement of menopausal symptoms.
*Rheum palmatum* L.	Rhizome	ND	2 (2%)	ND	*Radix, rhizoma*: Digestive function. Regularity of intestinal transit.
*Taraxacum officinale* (L.) Weber ex F.H.Wigg	Root	Liquid integral extract	2 (2%)	ND	*Herba* cum *radicibus, radix*: Hepatic functionality. Digestive function. Regularity of the intestinal transit. Detoxification of the organism. Body fluids drainage.
*Trigonella foenum graecum* L.	Seed	Dry extract, powder	2 (2%)	Saponins	*Semen*: Triglycerides and cholesterol metabolism. Carbohydrate metabolism. Digestive function. Emollient and soothing effect (digestive system).
*Viscum album* L.	Aerial parts	Liquid integral extract	2 (2%)	ND	*Folium, herba*: Lipid metabolism. Antioxidant.
*Acacia senegal* (L.) Willd.	Gum (exudate)	Powder	1 (1%)	ND	*Gummi*: Emollient and soothing effect (digestive system). Carbohydrate metabolism. Cholesterol metabolism. Prebiotic: Intestinal flora balance.
*Adansonia digitata* L.	Seed	ND	1 (1%)	ND	*Cortex, folium, fructus, radix, semen*: Joint function. Improvement of menstrual cycle disorders. Improvement of menopausal symptoms. Supportive and restorative effect. Boosting the immune system. Regularity of blood pressure. Regularity of the intestinal transit. Functionality of the upper respiratory tract.
*Astragalus microcephalus* Willd.	Root	Dry extract	1 (1%)	Polysaccharides, 70%	*Radix*: Cholesterol metabolism. Boosting the immune system.
*Bambusa spp*.	Sprouts	Dry extract	1 (1%)	Silicium, 75%	*Germen*: Digestive function. Intestinal gas elimination. Nails and hair wellness.
*Berberis vulgaris* L.	Root bark	Dry extract	1 (1%)	Berberine, 98%	*Cortex ex radicibus*: Digestive function. Hepatic functionality. Regularity of intestinal transit. Functionality of the digestive system.
*Beta vulgaris* L.	ND	ND	1 (1%)	Trimethyglycine (betaine)^f^	*Folium, radix*: Antioxidant.
*Brassica oleracea* L.	ND	Juice powder	1 (1%)	ND	*Folium, flos*: Antioxidant. Regular functionality of the cardiovascular system. Digestive function. Joint functionality.
*Buglossoides arvensis* (L.) I. M. Johnst	Seed	Oil	1 (1%)	Omega‐3, 60% Omega‐6, 15% Omega‐9, 6%	ND
*Capsicum spp*.	ND	Powder	1 (1%)	ND	*Fructus, oleum‐resina*: Digestive function. Regular gastrointestinal motility and gas elimination. Regular functionality of the cardiovascular system. Normal blood circulation. Metabolism stimulation. Antioxidant.
*Centella asiatica* (L.) Urb.	Leaf	Dry extract	1 (1%)	ND	*Folium, herba*: Fight cellulite imperfections. Microcirculation functionality (heavy legs). Memory and cognitive function.
*Citrus Limon* (L.) Osbeck.	ND	Essential oil	1 (1%)	ND	*Pericarpum, aetheroleum*: Digestive function. Regular gastrointestinal motility and gas elimination.
*Citrus sinensis* (L.) Osbeck.	Fruit	ND	1 (1%)	Hesperidin^f^	*Pericarpum, aetheroleum*: Digestive function. Intestinal gas elimination.
*Coffea arabica* L.	Seed	Dry extract	1 (1%)	Chlorogenic acid, 45%	*Semen*: Tonic and metabolic support action. Antioxidant.
*Cucumis melo* L.	Fruit	ND	1 (1%)	ND	*Fructus*: Digestive function. Skin regeneration.
*Cyclanthera pedata* (L.) Schrad.	Fruit	ND	1 (1%)	ND	*Fructus*: Regularity of blood pressure. Carbohydrate and cholesterol metabolism. Digestive function. Mucose wellness and regeneration. Body fluids drainage. Functionality of the urinary tract.
*Desmodium adscendens* (Sw.) DC.	Leaf	Dry extract	1 (1%)	ND	*Folium*: Functionality of the upper respiratory tract. Joint functionality. Hepatic functionality.
*Echinacea purpurea* (L.) Moench.	ND	Dry extract	1 (1%)	Polyphenols, 4%	*Herba, radix*: Boosting the immune system. Functionality of the urinary tract. Functionality of the upper respiratory tract.
*Elaeis guineensis* Jacq.	ND	Oleum dry extract	1 (1%)	Tocotrienols, 21%	*Fructus, oleum*: Joint functionality. Sedative effect. Body fluids drainage. Functionality of the urinary tract.
*Fagopyrum esculentum* Moench.	Fruit	ND	1 (1%)	B_1_, 0.31 mg B_2_, 0.5 mg B_3_, 5.4 mg B_5_, 2 mg B_6_, 0.31 mg Folic acid, 68 mcg B_12_, 0.6 mcg Biotin, 16.5 mcg	*Folium, flos*: Functionality of the microcirculation. Functionality of the venous circulation. Regular functionality of the cardiovascular system. Regularity of blood pressure.
*Foeniculum vulgare* Mill.	Fruit	ND	1 (1%)	ND	*Fructus, aetheroleum*: Digestive function. Regular gastrointestinal motility and gas elimination. Improvement of menstrual cycle disorders. Body fluids drainage. Bronchial secretions fluidity.
*Galium aparine* L.	Aerial parts	Liquid integral extract	1 (1%)	ND	*Flos, herba* cum *floribus, summitas*: Body fluids drainage and functionality of the urinary tract. Detoxification of the organism.
*Garcinia mangostana* L.	Fruit pulp	Dry extract	1 (1%)	Mangostins, 40%	*Pulpa fructus*: Antioxidant. Boosting the immune system.
*Juglans regia* L.	Fresh unripe fruits	Liquid integral extract	1 (1%)	ND	*Pericarpum, semen*: Digestive function. Detoxification of the organism. Regularity of the intestinal transit. Functionality of the digestive system. Integrity and functionality of cell membranes. Regular functionality of the cardiovascular system.
*Lespedeza capitata* Michx.	Leaf, whole plant	Dry extract	1 (1%)	Flavones^f^	*Folium, herba, summitas*: Body fluids drainage. Functionality of the urinary tract. Detoxification of organism. Regular functionality of the cardiovascular system. Lipid metabolism.
*Marrubium vulgare* L.	Aerial parts	Liquid integral extract	1 (1%)	ND	*Folium, herba* cum *floribus*: Bronchial secretions fluidity. Thermoregulation. Digestive function. Intestinal gas elimination.
*Marsdenia sylvestris* (Retz.) P.I. Forst *Gymnema sylvestre* (Retz) R.Br.^g^	Leaf	Dry extract	1 (1%)	Gymnemic acid, 25%	*Folium*: Carbohydrate and lipid metabolism. Appetite control.
*Opuntia ficus‐indica* (L.) Mill.	Cladodes	Powder	1 (1%)	ND	*Cladodium*: Body weight balance. Modulation/limitation of nutrients absorption. Emollient and lenitive effect (digestive system). Regularity of the intestinal transit.
*Peumus boldus* Molina	Leaf	Liquid integral extract	1 (1%)	ND	*Folium*: Digestive function. Hepatic functionality. Body fluids drainage. Functionality of the urinary tract. Regularity of the intestinal transit.
*Raphanus sativus* L.	ND	ND	1 (1%)	ND	*Semen, radix*: Digestive function. Body fluids drainage. Functionality of the urinary tract. Bronchial secretions fluidity. Antioxidant.
*Rhamnus purshiana* DC. *Frangula purshiana* Cooper	Bark	Dry extract	1 (1%)	Cascaroside A, 18–20%	*Cortex*: Regularity of the intestinal transit. Digestive function.
*Rosa canina* L.	ND	Dry extract	1 (1%)	ND	*Fructus, falso fructus*: Supportive and restorative effect. Regularity of the intestinal transit. Antioxidant.
*Salix alba* L.	Bark	Dry extract	1 (1%)	Salicin, 20%	*Cortex, cortex ex ramis, folium*: Joint functionality. Thermoregulation. Sedative effect.
*Salvia miltiorrhiza* Bunge	Root	Dry extract	1 (1%)	ND	*Radix*: Functionality of the cardiovascular system. Regularity of blood pressure. Improvement of the menstrual cycle disorders. Antioxidant.
*Salvia hispanica*	Seed	Oil	1 (1%)	ND	Not included^i^
*Schizochytrium spp*.	ND	Oil	1 (1%)	ND	Not included^i^
*Spirulina platensis* (Gomont) Geitler	ND	ND	1 (1%)	ND	*Thallus*: Supportive and restorative effect.
*Triticum aestivum* L.	ND	Oil	1 (1%)	ND	*Fructus*: Source of carbohydrate and gluten proteins. *Fructus germinatus* (malt): Supportive and restorative effect. *Oleum germinis*: Antioxidant. Lipid metabolism.
*Vaccinium myrtillus* L.	Fruit	Dry extract	1 (1%)	Anthocyanosides, 1%	*Fructus*: Functionality of the microcirculation (heavy legs). Antioxidant. Ocular health. Regularity of the intestinal transit.
*Zingiber officinale* Rosc.	Rhizome	ND	1 (1%)	ND	*Rhizome, aetheroleum*: Digestive function. Regular gastrointestinal motility and gas elimination. Antinausea. Regular functionality of the cardiovascular system. Normal blood circulation. Joint functionality. Sedative effect. Improvement of menstrual cycle disorders.

^a^ Plant species are in decreasing order of prevalence in the commercial supplements and in alphabetical suborder.

^b^ The plant material used and the type of the extract, only when reported in the label.

^c^ Plant prevalence calculated as a percentage of commercial supplements that contain it (No./113×100).

^d^ Usable, pending definitions of claims about botanicals (Italian Ministry of Health, 2019b). Only physiological indications for plant materials found in the commercial supplements, are reported in the table.

^e^ According to the Italian MoH, it is included as “other nutrients and other substances with a nutritional and physiological effect” (Italian Ministry of Health, 2019c).

^f^ Marker reported in the label without its titration percentage.

^g^ Synonym of the botanical name.

^h^ Not determined.

^i^ Plants not included in the plant list annex because authorized as novel foods according to the Regulation (EU) 2017/2470 establishing the list of novel foods, in accordance with Regulation (EU) 2015/2283 on novel foods (European Commission, 2017).

According to the Ministerial guidelines, 23% (*n* = 16) of the plant species found in commercial dyslipidemia‐HDS have claims for affecting cholesterol/triglycerides/lipids metabolism, while 18% (*n* = 13) claims the improvement of the cardiovascular system. 51% (*n* = 36) of the plant species refers to other physiological effects. A small percentage either (4%, *n* = 3) does not have any claim at all, or (4%, *n* = 3) is not included in the list because clustered as “other nutrients and other substances with a nutritional and physiological effect”/novel food (i.e., RYR, *Salvia hispanica, Schizochytrium spp*.).

Other plant‐derived ingredients, as pure substances or extracts, were identified in the labels of dyslipidemia‐HDS; their frequency was as follows: policosanols (*n* = 11), resveratrol (*n* = 6), phytosterols (*n* = 5), astaxanthine, gamma‐oryzanol (*n* = 4), linear aliphatic alcohols (*n* = 3), beta‐sitosterol, quercetin, sterolic esters (*n* = 2), beta‐carotene, betaine (trimethylglycine), citroflavonoids, lutein, octacosanol, orange oil, polyphenols, red orange complex, and enzymax® (fermented maltodextrin) (*n* = 1).

The occurrence of additional nonbotanical ingredients is the following in decreasing order: coenzyme Q_10_ (*n* = 40), folic acid (*n* = 34), vitamin B12 (cyanocobalamin) (*n* = 16), vitamin B6 (pyridoxine) (*n* = 15), vitamin E (tocopherol) (*n* = 14), chromium (*n* = 13), vitamin B3 (niacin) (*n* = 12), omega 3 (EPA&DHA), zinc (*n* = 6), alpha lipoic acid, choline (*n* = 5), selenium, vitamin B1 (thiamine), vitamin C (ascorbic acid) (*n* = 4), inositol (myo‐), L‐carnitine, vitamin D3 (cholecalciferol) (*n* = 3), vitamin B2 (riboflavin), vitamin B5 (pantothenic acid), *Lactobacillus plantarum* (*n* = 2), chitosan, *Bifidobacterium bifidum*, freeze‐dried pork liver, L‐arginine, *Lactobacillus acidophilus*, *Bifidobacterium longum*, L‐theanine, N‐acetyl cysteine, S‐adenosyl methionine, vitamin B8 (biotin), and vitamin K2 (menaquinone) (*n* = 1).

### 3.4 Reports from Phytovigilance

From October 1, 2018 to 31 September 2020, 16 spontaneous reports of suspected ARs were collected by the Italian Phytovigilance System. Only 15 of them were considered because related to HDS (one contained only niacin). The frequency of reporting, in decreasing order, of each dyslipidemia‐HDS is the following: Armolipid plus® (27%, *n* = 4); Colesia oral gel® or Colesia® (27%, *n* = 4); Calip plus®, Cardiolipid®, Berbered plus®, Midaqor®, SlimMetabol®, RYR, and Colestà® (7%, *n* = 1).

The recurrence of the plant species and isolated botanical ingredients was as follows: *Oryza sativa* fermented with *Monascus purpureus* (*n* = 8); *Olea europea* (*n* = 3); *Berberis aristata*; *Curcuma longa*; sterolic esters; *Haematococcus pluvialis (n = 2)*; others such as: *Moringa oleifera*, *Polygonum cuspidatum*, *Malus pumila*, *Saccharum officinarum*, *Punica granatum*, *Vitis vinifera*, *Citrus bergamia*, etc. (*n* = 1).

The patients involved in the suspected ARs were 13 women and 2 men, 50–81 years aged (median age 63.35 ± 8.13). Particularly, the median age for men was 75.5 ± 5.5 years, while for women 61.3 ± 6.6 years.

Acute liver failure was reported in one case, where the death of a 71‐year old woman occurred. The patient took a HDS called SlimMetabol® in concomitance with the medicine Lobivon®. The outcome of the other patients was: 6 recovers (40%), 6 in progress of recovery (40%), 1 not recovered (7%), and 2 not reported (not‐known) (13%). All the data refer to the moment of reporting the suspected ARs.

The ARs reported (*n* = 33) were: asthenia (*n* = 2); joint, abdominal, muscles pain (10 reports); vomiting (5 reports); nausea (3 reports); erythema and hematological disorders (4 reports); rhabdomyolysis (3 reports); hepatitis (2 reports, of which one lethal); other ARs like cramps, body weight loss, hyperkalemia, etc. (11 reports).

A “positive” dechallenge was reported in 11 cases; in the remaining reports, information was not reported. Although the doses have not been reported correctly, the considered HDS appear to have been taken appropriately without any abuse. The reporters were healthcare professionals (10), patients (2), and manufacturers (3).

## 4 DISCUSSION

To the best of our knowledge, this is the first study that gives an overview, although preliminary, of sales data on HDS. It is a different approach that reflects the existing market and consumer practice on dyslipidemia‐HDS, as well as the challenges to be faced by scientific authorities and regulatory agencies when dealing with the complex correlation between herbal‐derived food supplements and human health.

This study describes the characteristics of commercial dyslipidemia‐HDS referring to their sales data, heterogeneity, labeling, claims, and spontaneous reports of suspected ARs.

From our data collection, a constant interest in herbal supplements for the management of cardiovascular risk was found, despite the lockdown period due to COVID‐19 pandemic. The number of new formulations of dyslipidemia‐HDS using various plant species has increased from 1 year to the other. A complexity in the formulations of the supplements marketed subsists.

The results evidence scarce standardization in terms of accuracy about plant species, plant material, type of extract, dose of extract, and percentage of active ingredient. In particular, the plant material is not reported in the label of all supplements. Although the dosages are reported for all the ingredients, the standardization lacks in a lot of dyslipidemia‐HDS; even when reported, the composition in terms of percentage of the markers (when known) is extremely variable. Moreover, some labels report the active ingredients and/or phytochemicals without their percentage (Table 2, footnote a); some others describe only the kind of phytoextract (Table 2, footnote b). We can find even the extract with the relative percentage but missing the pharmacological marker (Arteractiva®, black pepper 95%, despite the marker of black pepper is well‐known). In our study, markers of some plant extracts (i.e., *C. longa*, *Camellia sinensis*, *O. europea*) have a unique percentage of titration instead of a range. It could be intended as a harmonized standardization, but on the other hand, not all the labels report it. One supplement (Kolestina 10 complex®) has even an erratum standardization relative to monacolin K on its label reported by the producer website. In every supplement, there is at least one lacking element (the name of the species, the plant material used, the type of the extract, the percentage of the titration) that makes it difficult to evaluate the quality and therefore the safety profile of it.

There are also patented extracts (Stilvid®, Centellin®, Extramel®, Biox save®, Bergavit®) whose composition is not reported specifically either in the label or in the package leaflet or the website of the producer; this hinders the assurance of quality and a clear information for the consumers and healthcare professionals.

Two supplements (LFP colesttab® 5 mg, LFP colesttab® 10 mg) are difficult to be distinguished from each other, as having the same brand name and almost the same packaging, though different dosage. Frequently, the same producer markets many dyslipidemia‐HDS differing for only one ingredient, or the dosage of a single ingredient, or the pharmaceutical form. It could be strongly indicative of the prevalence of commercial rather than health interest/goals, as neither the efficacy nor the therapeutic effect could make the difference between them. Moreover, it could mislead the consumer. The producer approach ponders the benefit of more supplements with same claims marketed but a doubtful rationale of use rather than one standardized and fully studied one. It seems that “playing” with the complexity of ingredients (generally multicomponent supplements often with unpredictable effects) has become a common marketing strategy.

The websites of the producers often do not show publicly the labels. Healthcare professionals who require information must apply for registration. Considering that internet is a very important tool where the population frequently addresses its needs, a such practice does not support informed choice and consumers protection.

From a regulatory point of view, our study is part of a complex context. Indeed in Italy, in order to place on the market a dietary supplement, the business operator (manufacturer) must follow for each supplement the electronic notification procedure called “tacit consent” addressed to the MoH (Italian Ministry of Health, 2004, 2018, 2021). Accordingly, for HDS, the MoH assesses the compliance with the current legislation (also implemented by annex 1 of the Directorial Decree of August 4, 2021) in order to guarantee the product safety and the appropriate information for consumers. In case of remarks and/or concerns about the label, the business operator has 30 days to respond to the MoH by adapting and/or modifying it meanwhile that the product is already on the market. The information related to the notified supplement must include the definition of the botanical, plant's characteristics, a copy of the label used for marketing, and other details reported in the MoH website (Italian Ministry of Health, 2004 ).

Therefore, the legislation represents the key point to strengthen the requirements for the business operators. For example, the Food and Drug Administration (FDA) requires that the label of a dietary supplement must include “a domestic address or domestic phone number” in order to enable the consumer to report adverse events associated with the use of it. In this context, should be highlighted that the FDA, as well as the MoH, are not authorized to review the safety and effectiveness of dietary supplements before they are marketed (FDA, 2019).

The MoH guidelines recommend some plant species for dyslipidemia that are not frequent in commercial supplements or vice versa. Furthermore, some of them are not at all supported by scientific literature. In this context, we were faced with three different situations:

1. plants that do not occur frequently in dyslipidemia‐HDS though having claims according Ministerial guidelines to improve the lipid profile (*Lagerstroemia speciosa, Plantago ovata, Glycine max, Trigonella foenum graecum, Cassia mimosoides var. nomame, Viscum album, Acacia senegal, Astragalus microcephalus, Cyclantera pedata, Lespedeza capitata, Gymnema sylvestre, Opuntia ficus‐indica, Triticum aestivum*). Scientific literature about the efficacy/safety of these botanicals is very scanty (weak). Preclinical data are often lacking and, when exist, composition and standardization of extracts are not specified (Ouzir, El Bairi, & Amzazi, 2016; Stohs, Miller, & Kaats, 2012). Despite the beneficial effects claimed, the few clinical trials performed have several limitations that require additional randomized clinical studies (Jovanovski et al., 2018).
2. plants that are quite frequent in the commercial supplements but do not have claims according to Ministerial guidelines (*C. bergamia, M. pumila cultivar. annurca, Buglossoides arvensis*). Scientific evidence is also quite inconclusive and/or insufficient (Kłosiewicz‐Latoszek, Cybulska, Stoś, & Tyszko, 2021; Tenore et al., 2017).
3. plants that do not have Ministerial claims specifically for dyslipidemia. Their claims often correspond to other indications of use of a single supplement (*S. officinarum*, *C. longa*, *P. cuspidatum*, *C. sinensis, H. pluvialis, V. vinifera*). These botanicals occur frequently in the dyslipidemia‐HDS as well as in scientific literature as agents for lowering plasma lipid levels, even though evidence is inconclusive and conflicting (Awad, Penson, & Banach, 2016; Woerdeman et al., 2017). Given that dyslipidemia ranks together with diabetes, obesity, and hypertension as a CVD risk factor, the effects of the above mentioned plant species could help in reducing CVD risks. Indeed, several studies confirm their antiinflammatory, antioxidant, and hypoglycemic effects (Ahmad, Alvi, Iqbal, & Khan, 2020; Qin et al., 2017; Ursoniu, Sahebkar, Serban, & Banach, 2015). Nevertheless, scientific literature highlights risk of toxicity that is, *C. sinensis* (Bedrood, Rameshrad, & Hosseinzadeh, 2018) and *C. longa* (Menniti‐Ippolito et al., 2020), in particular of extracts enriched in a specific phytoconstituent (e.g., curcumin, EGCG). The case of curcuma is particularly important, as this botanical is a best seller among others owing to a lot of health claims. Therefore, it gives the impression of being a “panacea,” but in contrast, it is subject of concerns from both regulators like MoH and evidence‐based research.

Our data suggest RYR as the most frequent botanical in dyslipidemia‐HDS and the most reported ingredient for suspected ARs, but it is also more studied in comparison to other plant species. Probably for all these reasons, it remains of higher concern for its safety profile. In different European countries like Germany, Austria, the use of HDS containing monacolin K is strongly recommended under medical supervision. In Switzerland, it is even prohibited in food supplements (German Federal Institute for Risk Assessment, 2020). In its latest opinion, EFSA considers monacolin K alone of risk for human health (EFSA, 2018). In our study, dosages of 1.5–10 mg of monacolin K are recommended in the dyslipidemia‐HDS. Moreover, monacolin K is frequently associated with other ingredients, which have not been fully studied together. According to the Italian MoH, RYR is not included as a botanical in the plant list annex (Italian Ministry of Health, 2019b), whereas, monacolin K is classified as “other nutrients and other substances with a nutritional and physiological effect” (Italian Ministry of Health, 2019c). In scientific literature, RYR is frequently defined as “nutraceutical” despite this term does not appear in the legislation. It is indicative of the fact that there is no compliance between regulatory framework, market, and scientific literature. The results remain difficult to be evaluated because the same plant/compound might fall in various categories such as that of novel food, botanicals, functional food, etc. Neither efficacy nor safety proofs are required. The first ones are not necessary, given that supplements could not claim any efficacy, while the safety profile is based only on the traditional use.

The coexistence of many botanical and heterogeneous nonbotanical ingredients could not support the risk/benefit profile of the supplement. Scientific literature describes some combinations that are sold in the market. *B. aristata* and *S. marianum* together (Berberol®) seem a good choice because of the P‐glycoprotein inhibitory effects of silymarin. As the poor oral bioavailability of berberine is due to the P‐glycoprotein‐mediated efflux mechanism, it can be improved by silymarin. Although in meta‐analysis, they appear safe and well‐tolerated as a combination, limited high‐quality clinical studies and large patient‐populations are underlined (Tóth et al., 2020). Armolipid plus®, that in our study, is one of the most sold supplements and most reported for suspected ARs, is also one of the most studied multi‐ingredient formulations. On the contrary, it comes out as a safe alternative in improving the lipids profile in clinical practice. Systematic literature describes no increased risk of neither musculoskeletal nor gastrointestinal disorders of the multi‐ingredient preparation made of berberine, policosanols, astaxanthin, monacolin, folic acid, and CoQ_10_ (Cicero et al., 2021). It is therefore difficult to recommend the supplement to the consumer. Levelipduo®, a combination of RYR, sterols, policosanols and niacin, has undergone to a randomized, double‐blinded, placebo‐controlled clinical study demonstrating a good tolerability besides the significant decrease of lipids plasma levels in mild hypercholesterolemia. However, relevant limitations regarding the number of enrolled subject, the short‐term duration are underlined. It seems that the combination between RYR and phytosterols could be similar to that of statins and ezetimibe as the mechanisms of actions are almost the same (Cicero, D'Addato, & Borghi, 2020). Accordingly, it would be important to further investigate its safety profile along with its efficacy. Other combinations of botanicals have been tested in clinical studies: RYR, artichoke and Banaba extract; RYR, artichoke and policosanols; RYR, policosanols and silymarin (Cicero et al., 2017). Their efficacy comes out with some positive results. Although the safety appears without concerns on the available data, the latter are not enough to draw conclusions. Almost all the combinations require long‐term randomized trials, large observational cohort studies, epidemiological studies.

Referring to the nonbotanical ingredients, some of them appear to have claims substantiated by EFSA. Niacin helps the maintenance of normal LDL‐C, HDL‐C and TGs (EFSA, 2009b). Folic acid can be beneficial in reducing the CVD risk because of its functional and regulatory role in the homocysteine metabolism (EFSA, 2009a). Although the risk of toxicity seems to be quite low, long‐term use of high doses could have important side effects (flushing, stomach irritation, liver toxicity). Therefore, the use should be under the control of a healthcare professional, considering all these multi‐ingredient formulations that a single individual can take for health benefits. The excessive intake of trace minerals (zinc, selenium) could be harmful in the CVD prevention (Mohammadifard et al., 2019). Therefore, the rationale of putting many ingredients together should be carefully evaluated.

All the HDS reported to the phytovigilance were used for lowering lipid levels, except the case of death in which the supplement was taken in order to improve body weight balance, cholesterol, and blood pressure. As the causality assessment was not performed, this report remains only suspect regardless the association between the supplement's intake and the AR. However, it should be noted that in the case of death, the product consists in a complex multi‐ingredient preparation (25 ingredients). This composition makes impossible the prediction of the final biological effect of the product. Furthermore, the industrial choice of a such composition should be justified in detail on the basis of public scientific studies. However, to date, no clinical safety evidence is required on the composition of the finished product, to be marketed as a food supplement.

In our study, we cannot compare directly the reports for suspected ARs with the dyslipidemia‐HDS sold because phytovigilance collects reports from all the Italian territory and our sales data concern only two pharmacies. This is a limitation of the study that does not allow us to address the sales volume of each single supplement reported. However, due to this limitation, a gap between the low number of reports and the wide range of dyslipidemia‐HDS marketed comes to attention. Hence, underreporting of suspected ARs about dyslipidemia‐HDS can be evidenced. Moreover, underreporting together with the extreme variability of commercial multi‐ingredient HDS makes it difficult to evaluate the impact of a single component independently. Taking into account the above safety considerations, a low awareness of the population about the safety of HDS appears obvious. Underreporting could be a signal for implementing programs of counseling and education in order to address the needs of the consumers and to protect their health. Further deeper investigation on sales data of dyslipidemia‐HDS of all the Italian territory pharmacies is required.

## 5 CONCLUSIONS

Overall, our findings point out the limited compliance of commercial dyslipidemia‐HDS and scientific research about them. What is placed on the market does not match with what does exist in scientific literature, in terms of methodological issues. Lack of a similar and constant biochemical composition of HDS does not support the reproducibility of the biological and pharmacological activities. Hence, the safety cannot be assured. Unmet needs can be underlined because of the lack of concordance between the use of multi‐ingredient supplements in real life and regulatory framework. It would not play therefore in favor of the safety profile of HDS.

This study could contribute to increase the importance of phytovigilance. The risks attributed to the use of dyslipidemia‐HDS should be highlighted in order to allow their rationale use, enhance their benefits, and prevent alarmism related to botanicals and/or supplements containing them.

Botanicals must be, therefore, appropriately characterized and defined; accuracy and controlled information must be assured in order to provide consumers with guidance about what they purchase and consume. Analyzing their label and properties that are claimed, knowledge on HDS would improve. We should never forget that supplements cannot (and must not) boast therapeutic or “miraculous” properties. Supplements can be used to maintain a physiological state of health; accordingly, they should at least guarantee their intrinsic safety.

## 6 RECOMMENDATIONS

*Addressed to manufacturers/business operators*

- Encourage simple compositions in order to avoid interactions, even within the same product.
- Enhance the marketing of standardized HDS in order to justify the recommended dose.
- Try to focus also on the sales of HDS via internet increasing the accuracy and transparency for consumers and healthcare professionals.

*Addressed to consumers*

- Paying attention to the label and rely on the counseling of a healthcare professional in order to choose the right supplement and to prevent herb–drug interactions.
- Be mindful that supplements do not have therapeutic activity but only adjuvant effects next to an adequate lifestyle, in particular a healthy diet.

*Addressed to regulators and policymakers*

- Establish science‐based public standards and prioritize them respect the traditional use for marketing authorization.
- Enhance surveillance programs/criteria by tracking more suspected ARs from HDS in order to increase the safety information for consumers.

## CONFLICT OF INTEREST

The authors declare no conflicts of interest.

## Data Availability

The data that support the findings of this study are available from the corresponding author upon reasonable request.
